# Epothilone B from *Aspergillus fumigatus* with a strong antiproliferative and anti-tubulin polymerizing activities; apoptosis, and cell cycle analyses

**DOI:** 10.1186/s12866-025-04086-1

**Published:** 2025-06-26

**Authors:** Ashraf S. A. El-Sayed, Safa W. Aziz, Ahmed H. Moustafa, Noha M. Saad, Mohamed Ali

**Affiliations:** 1 Enzymology and Fungal Biotechnology Lab, Botany and Microbiology Department, Faculty of Science, Zagazig University, Zagazig, 44519 Egypt; 2 Department of Laboratory and Clinical Sciences, College of Pharmacy, University of Babylon, Al-Hillah Babil, Iraq; 3 Chemistry Department, Faculty of Science, Zagazig University, Zagazig, 44519 Egypt; 4 Biochemistry Department, Faculty of Science, Zagazig University, Zagazig, 44519 Egypt; 5 Biochemistry Department, Faculty of Dentistry, Sinai University, Kantara, 41612 Egypt

**Keywords:** *Aspergillus fumigatus*, Epothilone B, LC–MS/MS analysis, Anticancer activity, Apoptosis, Cell cycle analysis

## Abstract

Epothilones were designated as one of the most recognized chemotherapeutic agents towards the drug-resistant tumors, for their higher potency to bind and stabilize the β-tubulin arrays, stopping the cell cycle. Epothilones were chemically resolved from *Aspergillus fumigatus* # MN744705.1, that being more affordable source than *Sorangium cellulosum*, for its rapid growth and unique biological behaviour. So, the aim of this work was to emphasize the chemical identity and efficacy of *Aspergillus fumigatus* Epothilone*.* The Epothilone structure of *A. fumigatus* was determined by HPLC, FT-IR, LC–MS analyses, with 507.7 m/z, compared to the authentic one of *S. cellulosum*. *Aspergillus fumigatus* epothilone B had the highest activity against HepG-2 (IC_50_ value 6.3 μM), and HCT-116 and Pc3 (IC_50_ value 7.4 μM), compared to Vero cells (18.7 μM) with selectivity index 2.9, 2.5, and 2.47, respectively. The anti-tubulin polymerizing potency of the purified Epothilone was about two folds more than Taxol, with an obvious resilient arrest to the cellular growth of the cells of HepG-2 at G2/M phase. The total, early and late apoptosis of the HepG2 cells were increased by 26.5%, 15.9% and 7.6%, respectively, with the epothilone of *A. fumigatus,* with an overall increase of apoptosis by 12 folds, compared to control. The caspase-9 and 3 activities were increased by 4 folds and 2.5 folds, with the Epothilone B, as revealed from the colorimetric activity and gene expression analyses. The level of released LDH of HepG-2 cells was increased exponentially with the Epothilone concentration, ensuring their negative effect on the plasma membrane permeability. From the docking results, the binding energy of Epothilone B with the tubulin-β was -9.96 kcal/mol, that was lower than Taxol (-7.87 kcal/mol), ensuring the higher affinity of Epothilone B to bind with the β-tubulin protein.

## Introduction

Epothilones are macrolides that were initially recovered by *Sorangium cellulosum,* displaying a wide activity against various tumor cells [1]. The antiproliferative activity of epothilones has been elaborated from the potency to bind with the microtubule arrays β-tubulin, during the cellular division causing an ultimate halt at G2/M phase [2, 3]. In 2007, the epothilone B “Ixabepilone” has been approved by FDA [4]. Epothilones and Taxol have the same biding sites on tubulin, stopping the tubulin disassembly, during the mitotic division of cellular growth [5]. The common antitumor drugs including paclitaxel, docetaxel and epothilones prevent the cellular growth by stabilizing the microtubules over-dimerization/destabilization of microtubules within the cell, stopping the cells at the G2-M phase [6]. These compounds change the dynamics of microtubule polymerization, causing mitotic arrest and eventually apoptosis of cancer cells [7, 8]. The tubulin targeting compounds were grouped into 2 classes; 1- Microtubule stabilizing compounds including Taxol, docetaxel, discodermolide, and epothilones. 2- Inhibitors of microtubule polymerization like vincristine, vinblastine, colchicines, cryptophycins, and combretastatins [9]. Practically, epothilones has a significant antitumor activity towards the different drug-resistant tumors, for its higher solubility in water, low binding energy with tubulin. The activity of epothilones towards various drug-resistant cells expressing the *P*-glycoprotein was a thousand fold higher than Taxol [5, 9].

Currently, epothilones production was employed from *S. cellulosum*, nevertheless, the predominantly slow growth is the restraining factor that halts the implementation of this approach industrially, making this compound is an extraordinarily costly [10]. Naturally, in submerged fermentation, *S. cellulosum* form a clump structures that impedes the physiological absorbance of nutrients and metabolites transportation, with a consequent decreasing to the secondary metabolites productivity [11]. Myxobacteria have a complex life paradigm by nourishing in groups, with emerged myxospores in cell density manner, with developed multicellular fruiting bodies [11]. Tubulin subunits “alpha and beta-tubulin”, combine to produce microtubules, a significant form of cytoskeletal filament in cells. So, searching for a novel alternative source for Epothilone production was the main challenge. The Epothilone productivity by *A. fumigatus* of identical structure and anticancer activity of *S. cellulosum* Epothilone opens a new avenue for production of this lead compound industrially [9, 12]. Therefore, the biological activity of *A. fumigatus* Epothilone was considered based on their cytotoxic activity, cell cycle, apoptosis analysis, anti-tubulin polymerizing activity, in addition to the molecular docking analysis.

## Materials and methods

### Fungal culture conditions

*Aspergillus fumigatus* of accession number MN744705.1, deposition number AUMC14078, as the potent Epothilone B producer, was selected from our previous studies [9, 12]. The fungus grown on potato dextrose broth (PDB) media (Cat.# DF0549.17.9), incubated at 29 ± 1.0 °C for 15 days, then the fungal cultures were amended with methylethylketone (MEK), re-incubated overnight at 200 rpm, and the entire cultures were homogenized in a mechanical blender for 10 min, filtered by sterile cheesecloth [9, 13, 14]. The layer of methyl ethyl ketone was separated from the aqueous layer by separating funnel, and the lower layer of solvent was evaporated, dissolved in of dichloromethane (1 ml). The sample was fractionated by TLC (Silica gel 60 F254) with dichloromethane: methanol (95:5) [9, 12]. The putative spots were detected at λ_254_ nm, compared to the standard ones (Cat#. 152,044–54-7). Epothilone was eluted from the silica particles, and re-suspended in dichloromethane, the putative compound was eluted for further chemical validation analyses [9, 12].

### HPLC, FT-IR and LC‑MS/MS

The purity of resolved epothilone was assessed by the HPLC with methanol: water (70:30 v/v) at flow rate 1.0 ml/min, for 25 min, and its concentration was verified from peak area at λ_249_ nm, paralleled to authentic one [15, 16]. The sample FTIR spectra were assessed at 400–4000 cm^−1^, and the shifts were expressed in ppm (δ-scale) and hertz (Hz) [9].

The sample identity was assessed by LC–MS/MS with electrospray source [9, 12], with acetonitrile in formic acid (0.1%), an compound was eluted at 0.2 ml/min flow rate, with ion trap from 300–2000 m/z in a positive mode. The identity of compound was assessed relied on their spectra by the NIST library.

### Antiproliferative and anti-tubulin polymerizing activities

The antiproliferative activity of Epothilone was estimated for liver (HepG-2), breast carcinoma (MCF7), prostate cancer cells (Pc3), and colon cancer cells (HCT 116) (purchased from ATCC), were assessed by MTT assay, paralleled to Vero cells [17], The plate was inoculated by 10^3^ cells/well in a 100 μl DMEM medium (with 10% FBS, 10 μg/ml insulin), incubated for 12 h at 37 °C, under standard conditions, then supplemented by the putative Epothilone, incubated for 48 h, then MTT solution was included and the plate was placed for 6 h, the absorbance of emerged complex was assessed at λ_570_ nm, and the IC_50_ values were determined [9].

The anti-tubulin polymerizing activity of epothilone was assessed using the cytoskeleton assay Kit (Cat. #BK011P) [12]. The tubulin proteins of Porcine brain (250 μl) were dispensed ice cold Tubulin Polymerization buffer, and the epothilone were added to the mixture at different concentration in 100 μl total volume in the 96-well plate, using Paclitaxel as positive controls (10 μM). The polymerization of tubulin was assessed by measuring the turbidity change at λ_340_nm, and the IC_50_ values were calculated by the concentration reducing the activity of tubulin polymerization by ~ 50%, compared to control.

### Cell cycle and apoptosis

The cell cycle was examined by propidium iodide with the purified epothilone by the flow cytometric assay (Cat#ab139418). The tumor cells were inoculated to a 48-well plate, incubated at 37 °C overnight, then epothilone was amended at its IC_25_ values, for two days, then the cells were collected by centrifugation. The harvested cells were fixed in ethanol, stained with the PI in the dark, then the DNA was determined at Ex λ_493_ and Em λ_636_ nm, and the stages of cell cycle were assessed [5, 12].

The cellular apoptotic processes were assessed with Annexin V-FITC Apoptosis (Catalog #: K101-25) [12], that subsequently form Annexin V-PS conjugates. The cells were inoculated to 96-well plate (3 × 10^5^ cells/well), amended with various epothilone concentrations, incubated for two days. After incubation under standard cultural conditions, the cells were harvested, then amended with buffer, and Annexin V-FITC, and PI, and then kept for 15 min at 25 °C, in dark. The conjugates of Annexin V-PS were measured at Ex λ_488_ nm and Em λ_530_ nm) with FITC signal detector.

### Caspase-3 and −9 activity

The activities of caspase-3 and 9 of the HepG-2 cells was assessed with DEVD-pNA and LEHD-pNA, respectively, as chromogenic substrates, liberating the p-nitroanilide (pNA), as detected at λ_405_ nm [18, 19]. The reaction mixture had 50 μl of enzyme extract, 10 mM DTT, and 4 mM DEVD-pNA or LEHD-pNA as substrates, in 100 μl total volume, for 60 min at 37 °C, and the absorbance was detected at λ_405_ nm.

### Assay of the reactive oxygen species (ROS)

The ROS of HepG-2 cells in response to Epothilone concentrations were determined by dichlorofluorescin diacetate (DCFH-DA) [20, 21]. In 96-well black plate, 10,000 cells per well were seeded, allow to adhere to the plate surface for one day at 37 °C incubated, then amended with epothilone (10–40 μg/ml), and re-incubated for 24 h. The plates were cleaned with HBSS, amended with 1 ml of DCFH-DA, incubated for 30 min at 37 °C. The cells were lysed, centrifuged for 10 min at 2500 rpm, and the concentration of ROS was determined from the fluorescence emission λ_520_ nm at excitation λ_485_ nm.

### Assay of lactate dehydrogenase leakage

The leakage of lactate dehydrogenase of HepG-2 cells with the Epothilone was assessed with the LDH-cytotoxicity assay kit [22]. The 96-well plate was inoculated with 20 × 10^3^ cells/well, in presence of Epothilone B (10–40 μg/ml) for 24 h, under standard cultural conditions. The plate was centrifuged at 2500 rpm for 5 min, and the LDH activity was determined according to the manufacturer’s instruction at λ_340_ nm, compared to the controls [22].

### Molecular docking

The docking study of the extracted Epothilone and tubulin (PDB code: 5 W3 J) was performed with the Molecular Operation Environment (MOE) program, with the merck molecular force field [23, 24].

### Statistical analysis

The current experiments were performed three biological replicates, the data were represented by the means ± SD, and the significance values were analyzed by One-Way ANOVA.

## Results

### Chemical validation of the *A*. *fumigatus* Epothilone

**Fig. 1:**
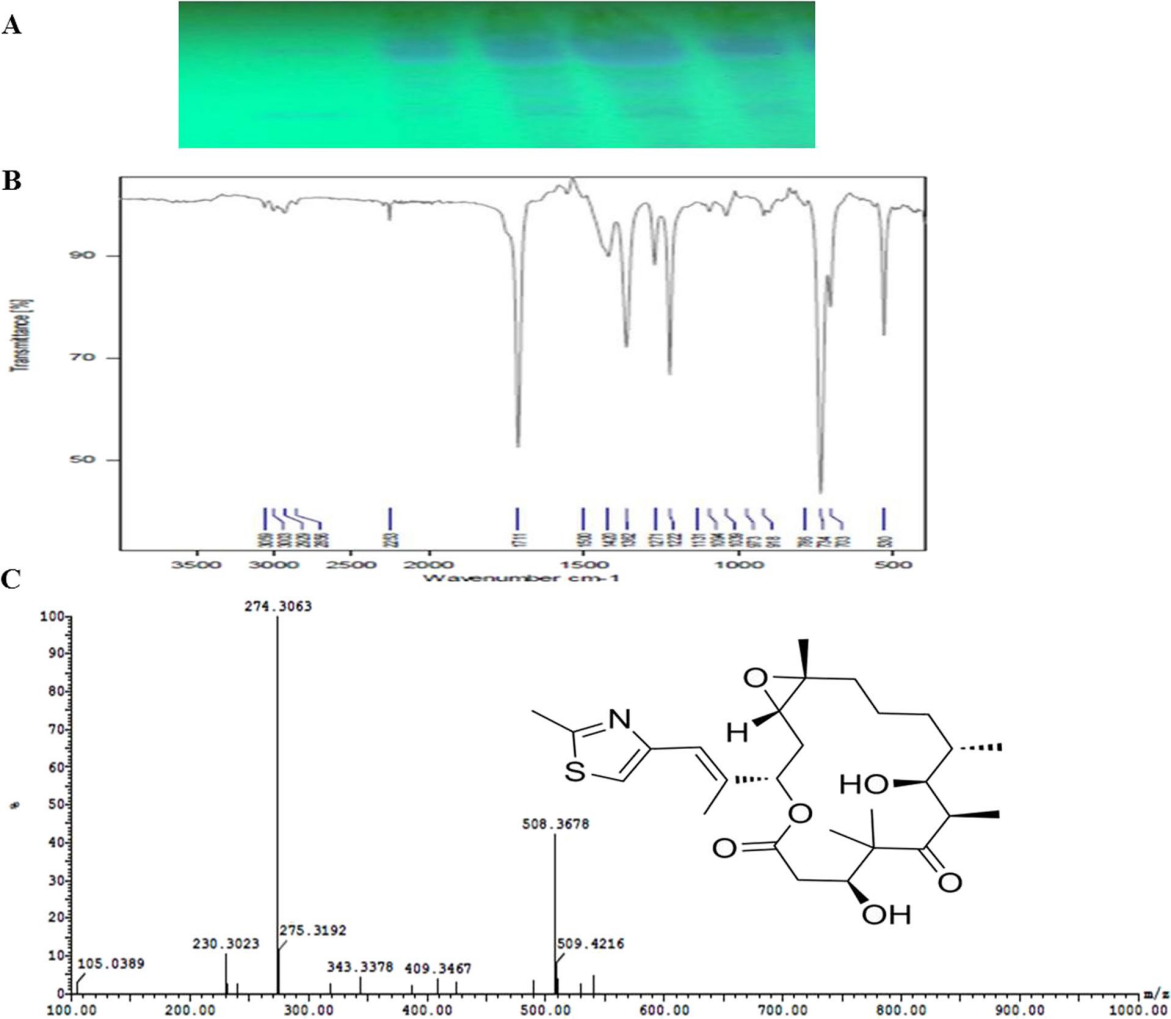
Chemical validation of the extracted epothilone B from *Aspergillus fumigatus*. **A** TLC of the extracted Epothilone from *A. fumigatus,* compared to the authentic one. **B** FT-IR spectra of the purified Epothilone. **C** LC–MS/MS spectra of the purified Epothilone with the onset chemical structure of Epothilone B

The isolate was cultured on PDB as aforementioned, then, epothilone was extracted and checked. From TLC chromatogram (Fig. 1), the sample had the identical mobility of the authentic, confirming the compound as Epothilone B. The spots of silica were scratched from the plate, the Epothilone was eluted, and its concentration was assessed by HPLC. The sample of *A. fumigatus* had the maximum absorbance at λ_235_ nm and same retention time of authentic one, as revealed from the UV-Spectra and HPLC analyses, respectively. The Epothilone B concentration of *A. fumigatus* was 60.1 μg/L. From the FT-IR spectra, the hydroxyl groups at 3300 cm^−1^, CO at 1663 cm^−1^ and epoxy ring at 1200 cm^−1^, were observed in Fig. 1. The peaks at 2921 cm^−1^, 1623 cm^−1^, 1485 cm^−1^, and 1099 cm^−1^ were consigned to the stretches of the aliphatic CH, ester groups and aromatic ring. Moreover, the structure of the sample was chemically committed from the LC–MS/MS, with 508.36 m*/z,* was identical to the molecular mass and fragmentation form of authentic one [25].

### Antiproliferative activity

**Fig. 2:**
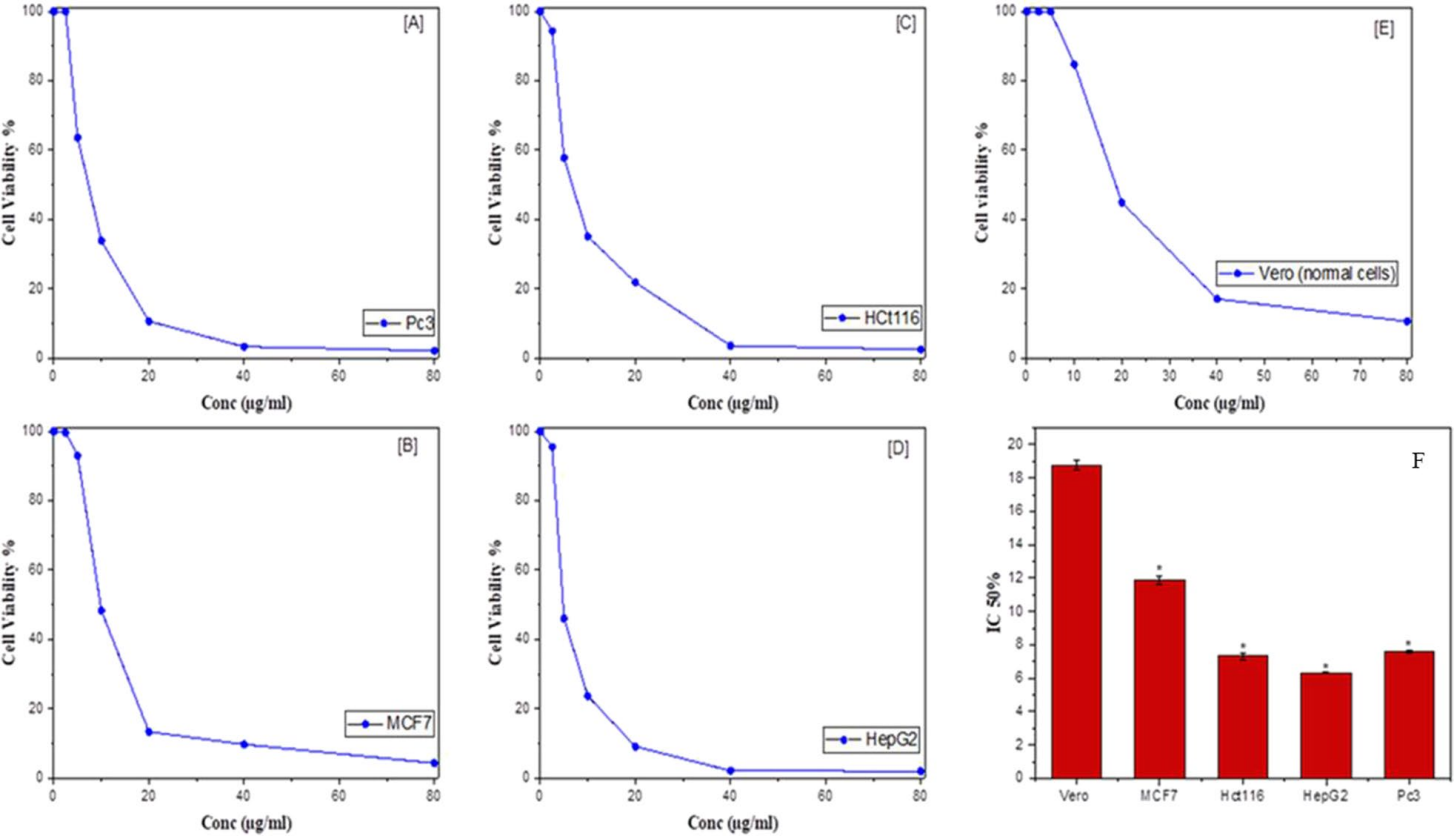
The cytotoxicity activity of the purified Epothilone B from *A. fumigatus* towards various tumor cells at concentrations (2.5–80 μg/ml). The viability of prostate cancer cells (Pc3) (**A**), breast cancer cells (MCF-7) (**B**), colon cancer cells (HCT 116) (**C**), and liver cancer cells (HepG2), normalized to Vero cells as control ones (**E**). The IC50 values of the Epothilone B towards the tested cell lines (**F**). *Significant difference as compared to the controls (*p* < 0.05)

The efficiency of *A. fumigatus* epothilone B was estimated for the different cells; HepG-2, MCF7, Pc3, and HCT116, normalized to Vero cells by MTT assay. *A. fumigatus* Epothilone has a considerable activity against the experimented cell lines in a concentration-dependent paradigm (Fig. 2). Practically, *A. fumigatus* Epothilone had the highest activity against the HepG-2 cells, followed by HCT116, Pc3 and MCF-7 cells, paralleled to the normal cells, as shown from the viability curves. From the IC_50_ values, the purified compound had the highest antiproliferative activity for the HepG-2 (6.32 ± 0.05 μM), followed by HCT 116 (7.34 ± 0.21 μM), Pc3 cells (7.6 ± 0.06 μM) and MCF-7 cells (11.91 ± 0.24 μM) compared to the Vero cells (18.77 ± 0.3 μM). From the IC_50_ values, the selectivity index of *A. fumigatus* Epothilone for HepG-2 cells, HCT-116 and Pc3 cells were 2.97, 2.56 and 2.47, respectively, paralleled to the Vero cells.

### Anti-tubulin polymerizing activity and cell cycle analysis

**Fig. 3:**
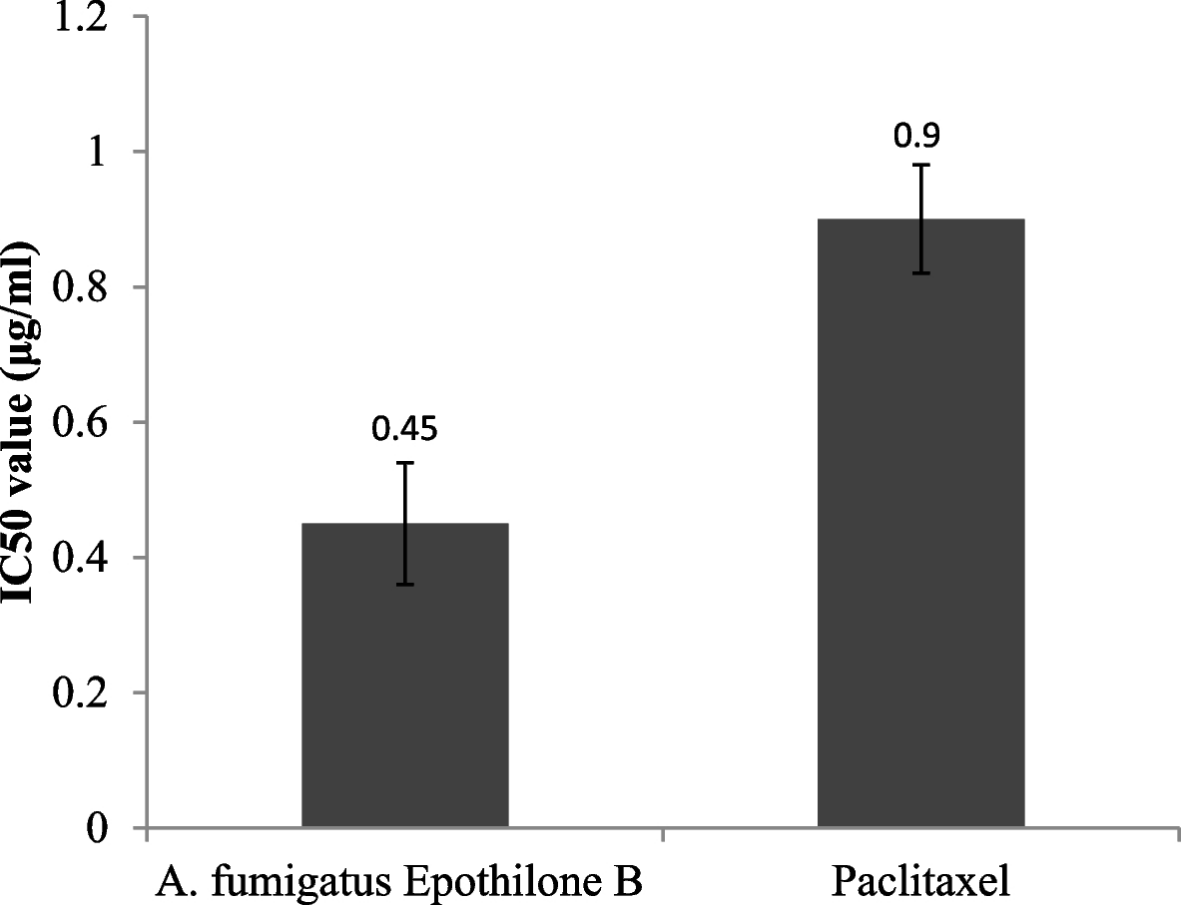
Anti-tubulin polymerizing activity of the extracted Epothilone B of *A. fumigatus*. *Significant difference as compared to the controls (*p* < 0.05)

The anti-tubulin polymerizing potency of epothilone was estimated by the fluorescence based assay. Epothilone was amended to the reaction at different concentration, and the tubulin polymerizing activity was measure by standard assay, compared to Paclitaxel as reference drug. From the data in Fig. 3, the IC_50_ value of the *A. fumigatus* Epothilone B was 0.45 μg/ml related to Paclitaxel.

**Fig. 4:**
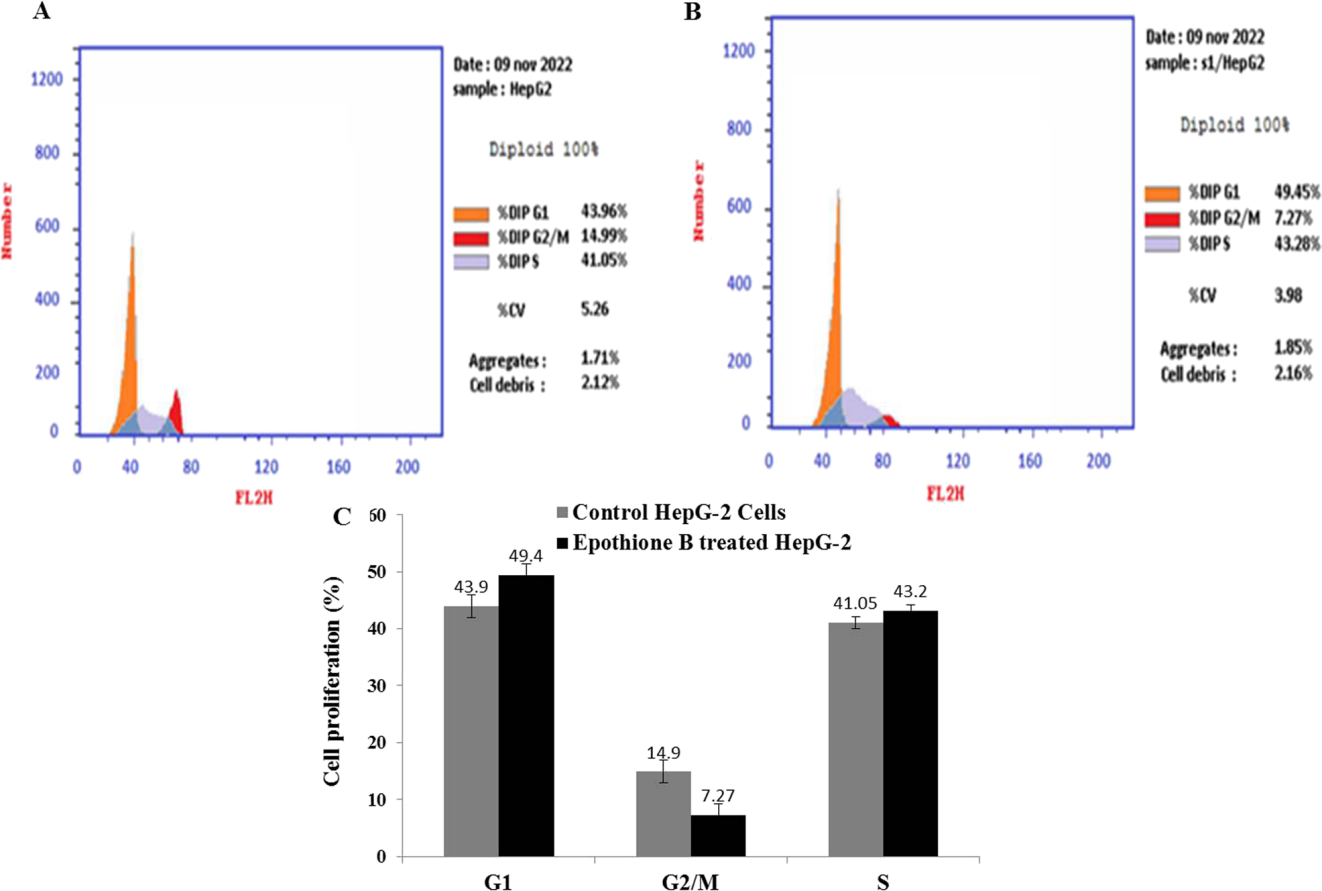
Cell cycle analysis of the HepG-2 cells in response to the extracted *A. fumigatus* Epothilone B, by the flow cytometry using the PI. The cell cycle analysis of the HepG-2 cells without Epothilone B (**A**), and in response to treatment with *A. fumigatus* epothilone (**B**). **C** The overall quantitative analysis of cell cycle. The values were represented by the means, followed by letters a, b within the same column that is a significantly different (ONE Way ANOVA, LSD test, *p* ≤ 0.05)

The cell cycle of the HepG-2 owing to *A. fumigatus* Epothilone was analyzed by PI, at its IC_25_ value, incubated for 24 h under regular conditions, and the cells were gathered, fixed by ethanol, the total DNA was flow cytometrically analyzed, the cell cycle phases were considered. The premier stop of the HepG-2 cells growth with epothilone of *A. fumigatus* was recorded at G2/M, paralleled to the normal cells (Fig. 4). The cellular proliferation of HepG-2 was reduced into 7.2%, in response to addition of the IC_25_ of Epothilone compared to normal cells (14.9%), revealing the competence of the compound to combat the cellular growth at mitotic stage G2/M phase. However, there the cellular growth phases G0-G1 and S phases of HepG-2 in response to Epothilone B and control was obviously similar, confirming the absence of cytotoxicity of the purified Epothilone on the cell cycles at these stages.

### Apoptosis analysis of HepG2

**Fig. 5:**
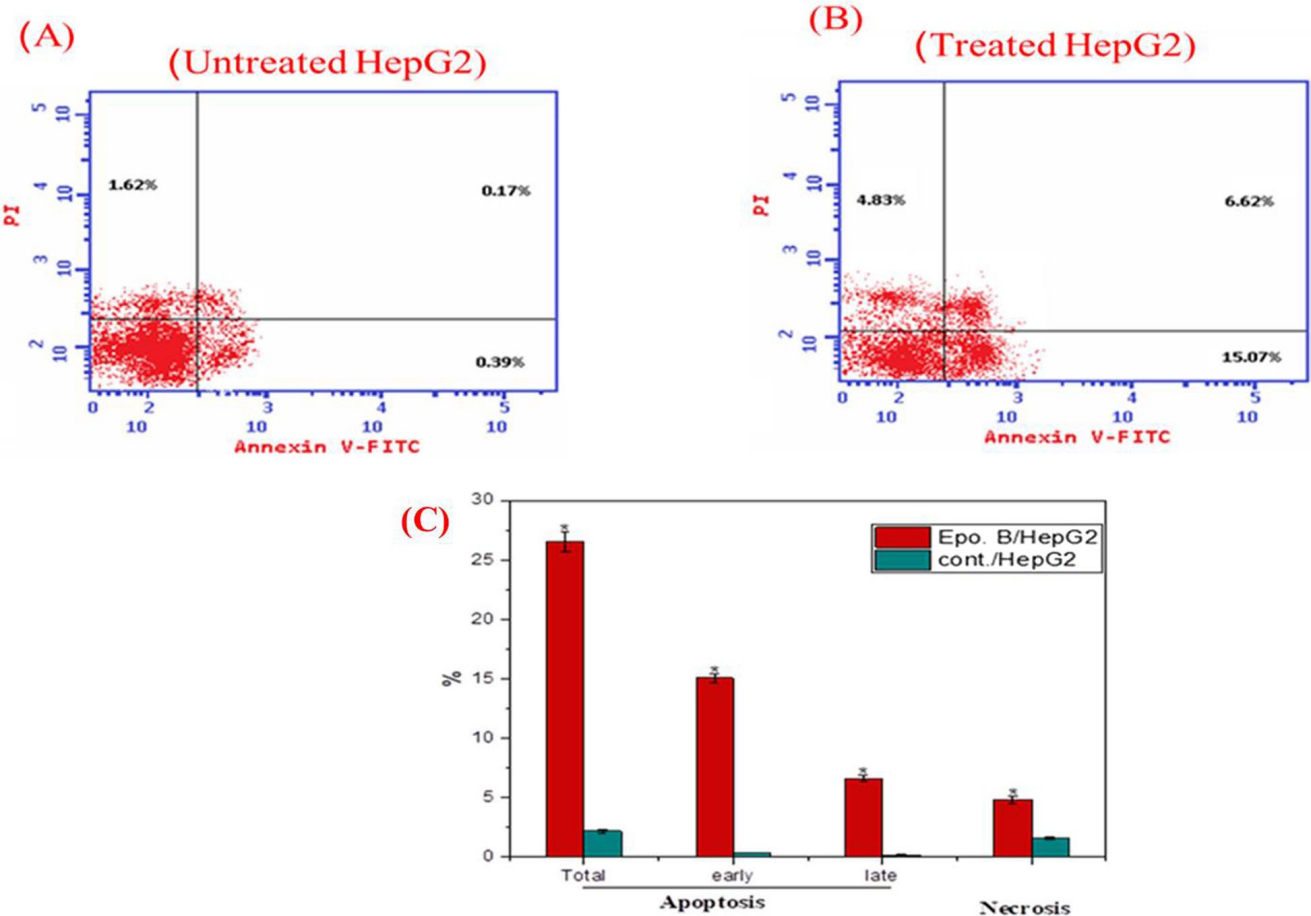
Apoptosis analysis by flow cytometry with Annexin V-FITC/PI dual stains of the HepG2 cells. The cells were treated with IC25 of *A. fumigatus* Epothilone B, the apoptosis was measured after 48 h of incubation. Apoptotic analysis of untreated HepG2-cells (**A**) and Epothilone B treated cells (**B**), and overall quantitative results of apoptosis (**C**). The results were statistically significant compared to the untreated cells controls (*p* < 0.05)

A substantial induction to the cells on the different apoptotic processes “early apoptosis, late apoptosis”, were recorded with the tested sample*,* paralleled to control (untreated) cells (Fig. 5)*.* The ratios of HepG2 cells in total, early and late apoptosis were 26.5, 15.9 and 7.6%, with *A. fumigatus* epothilone. However, the ratio of apoptosis of the control cells was about 2.1%, 0.5 and 0.2%, respectively. Thus, with *A. fumigatus* Epothilone, the total apoptosis was increased by twelve 12 folds, ensuring the biochemical significant effect of epothilone in inducing the cellular machinery of apoptosis. As well as, the ratio of necrosis of the normal HepG-2 cells was increased to about 5.1%, in presence of Epothilone, compared to the untreated cells (1.5%), ensuring to the cytotoxicity and specificity of the extracted Epothilone.

### Activity and molecular expression of caspase-3 and caspase-9 due to Epothilone B

**Fig. 6:**
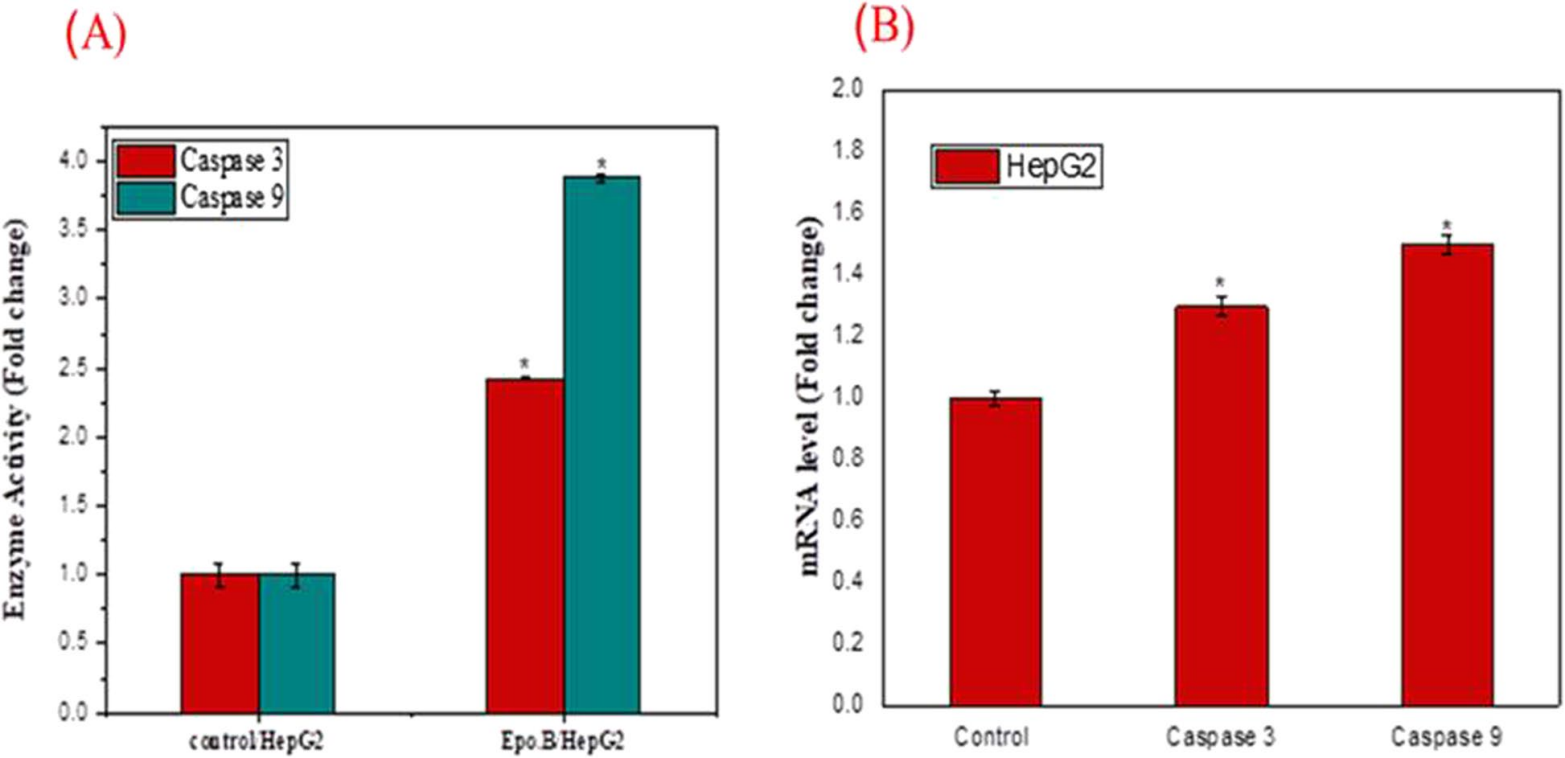
Activity of the caspase-3 and caspase-9 as an apoptotic response to the extracted *A. fumigatus* Epothilone B. The HepG-2 cells were grown at the IC25 value of the extracted Epothilone B, incubated for 24 h at standard conditions, then the activity of caspase-3 and caspase-9 was determined. **A** The activity of caspase-3 and caspase-9 in Epothilone treated HepG-2 and untreated cells by ELISA assay. **B** RT-qPCR analyses of caspase-3 and caspase-9 of HepG-2 cells treated with Epothilone B, compared to the untreated cells. The data were represented by the mean ± SD. The results were statically significant, compared to the control (*p* < 0.05)

The activity of caspase 3 and 9 of HepG-2 cells with the extracted Epothilone B was determined colorimetrically, in response to the IC_50_ of Epothilone (6.32 ± 0.05 μM). From the results (Fig. 6), the colorimetric activity of caspas-3 and 9 was dramatically augmented with the Epothilone B, related to the control cells. The efficiency of caspase-9 and 3 was strongly increased by 4 folds and 2.5 folds, respectively, in presence of Epothilone B, related to the control at *p* < 0.05.

The molecular expression of the caspase-3 and 9 was verified by RT-qPCR, using mRNA as template. From qPCR results (Fig. 6), the expression of caspase-3 and 9 was dramatically improved by 1.3 and 1.5 folds, respectively, related to the control. So, the colorimetric activity of caspase-3 and 9 and their molecular expression analyses by qPCR were clearly matched, ensuring the induction of the caspase-3 and caspase-9 with *A. fumigatus* Epothilone B.

### Influence of *A*. *fumigatus* Epothilone B on ROS generation and LDH leakage of HepG-2 cells

**Fig. 7:**
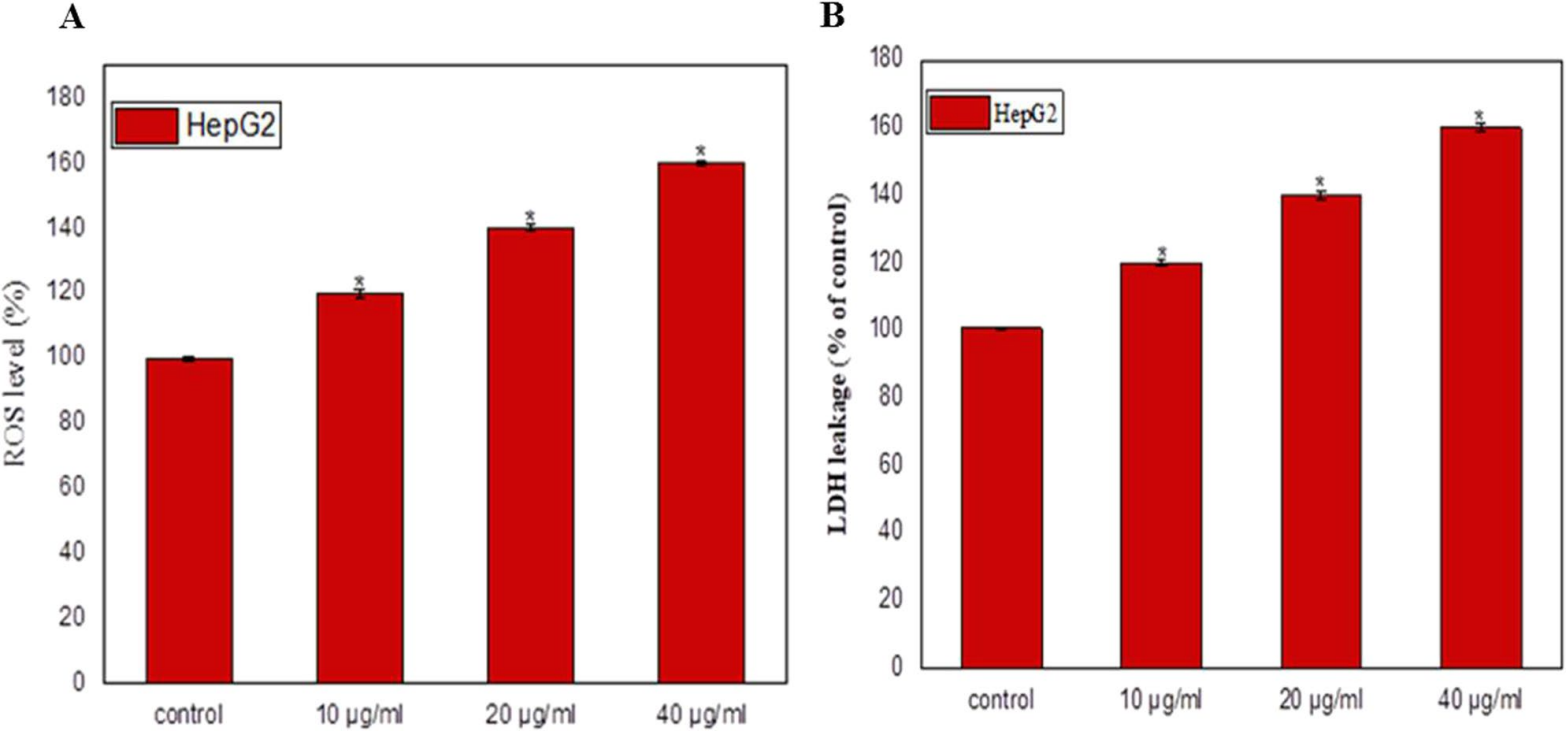
Generation of ROS and LDH leakage of HepG-2 in response to Epothilone B treatment. The HepG-2 cells were treated with *A. fumigatus* Epothilone B at different concentrations, incubated for 24 h at the standard conditions. The percentage of generated ROS of HepG-2 (**A**), and LDH leakage (**B**) in response to different concentrations of Epothilone B, compared to control. *Significant difference as compared to the control (*p* < 0.05)

The ROS generation has been used frequently as marker for numerous biological processes, such as inflammation, DNA damage and apoptosis. The impact of *A. fumigatus* Epothilone B on generating the ROS in HepG-2 cells was assessed, by amendment different concentrations of Epothilone (0, 0.1, 0.2 and 0.4 μg/ml) to the medium of HepG-2 cells, then measuring the ROS generation. From the results (Fig. 7), an obvious increase on the generation of ROS level with Epothilone concentration, in a concentration-dependent paradigm. The ROS level was increased by about 30% and 40% in response to 0.2—0.4 μg/ml of Epothilone, over the control (without Epothilone). So, Epothilone B from *A. fumigatus* had the potency to induce the ROS generation in HepG2 cells.

The influence of Epothilone B of *A. fumigatus* on activity of cytosolic LDH that responsible for converting lactate to pyruvate in HepG-2 cells, were assessed. Practically, with the presence of cytotoxic compounds, the LDH was diffused-out into the media due to the partial rupture of the plasma membrane. So, the upper activity of LDH in media reveals higher cytotoxic effect of extracted epothilone B. The levels of diffused out LDH was increased exponentially with Epothilone in a concentration-dependent paradigm (Fig. 7). The maximum percentage of LDH leakage was observed at 0.4 μ/ml, by ~ 50% over control, ensuring the negative consequence of epothilone on the plasma membrane permeability of HepG-2 cells.

### Molecular docking analysis

**Fig. 8:**
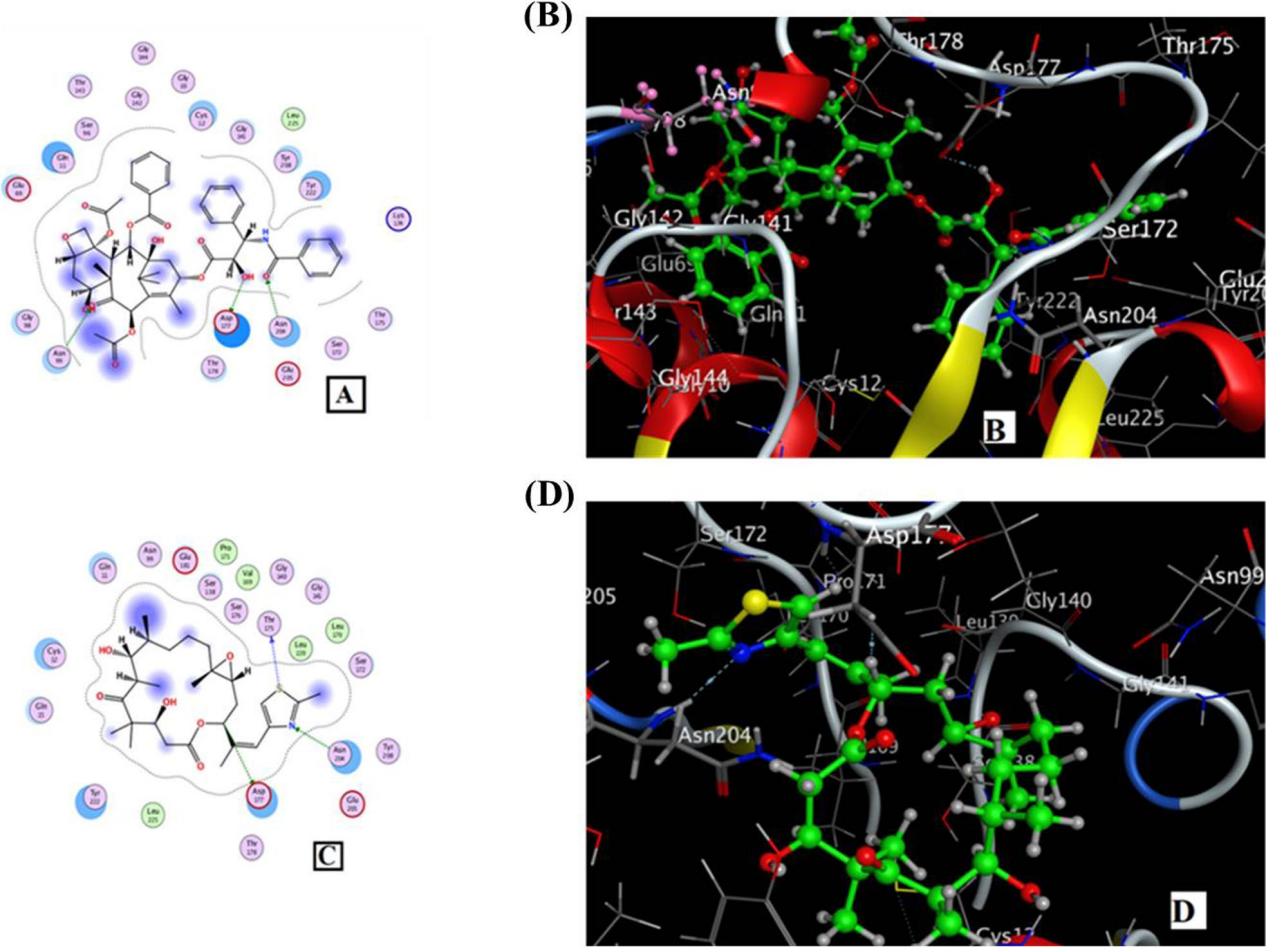
Molecular docking of β-tubulin with Epothilone, compared to taxol, as reference one. **A**, **B** 2D and 3D interaction of Taxol with the β-tubulin. **C**, **D** 2D and 3D interaction of Epothilone B with β-tubulin

Docking analysis was carried out to understand the interaction of the compounds with the target protein. The crystal structure of tubulin (PDB code: 5 W3 J) was retrieved from the Protein Data Bank [26]. The in silico modelling analysis of the purified Epothilone B was conducted with the beta tubulin binding sites (5 W3 J), compared to Taxol, as reference drug with the same mode of action and binding sites. The interaction parameters of Epothilone B and Taxol with tubulin-β (PDB code: 5 W3 J) were summarized in Table 1. The results revealed that Taxol powerfully verified the interaction of the target ligand sites by forming three H-Bonds: O (52), formed with the target protein via asparagine (Asn204), O(48) with Aspartic Acid (Asp 177) and O (25) with the asparagine (Asn 99) amino acid. However, hydrogen bonds were the common type of interaction, compared to the hydrophobic (Arene-H) bonding, as revealed from the binding energy illustrated in Fig. 8A, B. The energy of binding was calculated to be −7.87 kcal/mol. The docking result of the Epothilone-B showed three hydrogen bonds: S (30) with Threonine (Thr 175) amino acid, N (32) with asparagine (Asn 204), and C (11) with aspartic Acid (Asp 177) of the target protein (Fig. 8C, D). The binding energy of Epothilone-B with tubulin-β protein was −9.96 kcal/mol, that was higher than Taxol (−7.87 kcal/mol) (Table 1). The interaction sites of Epothilone-B were similar to the anti-cancer drug Taxol. The previous results of Epothilone-B showed a little better inhibitory effect on tubulin-β than Taxol.

**Table 1:** Apparent interaction parameters of the Epothilone-B and Taxol with tubulin-β (PDB code: 5 W3 J)

Ligand	Ligand sites	Receptor sites	Type of the interaction	Distance of bond (Å)	Binding energy (kcal/mol)	Total free binding energy (kcal/mol)
Taxol	O (52)	Asn 204	Side chain acceptor (H-Acceptor)	2.93	−1.2	−7.87
	O (48)	Asp 177	Side chain donor (H-donor)	2.94	−1.3	
	O (25)	Asn 99	Side chain acceptor (H-Acceptor)	3.02	−1.8	
Epothilone-B	S (30)	Thr 175	Backbone donor (H-donor)	3.64	−0.2	−9.96
	N (32)	Asn 228	Side chain acceptor (H-Acceptor)	3.4	−0.9	
	C (11)	Asp 177	Side chain donor (H-donor)	3.46	0.7

## Discussion

Epothilones are macrolides produced by the myxobacterium *S. cellulosum,* with strong potential anticancer activity against variety of multiple drug-resistant malignancies [27, 28]. The activity of epothilone was derived from its potency to form a strong binding with the cancer cells microtubule arrays, stopping the cellular division at G2-M phase [29]. The affordable feature of Epothilone is the higher water solubility, unique pharmacokinetic and bioavailability properties as well as their ability to inhibit cell lines resistant to paclitaxel [30]. The pharmacokinetic properties of epothilones were noticeably more affordable than Taxol, by binding to certain loci on the tubulin surface supporting tubulin polymerization [31]. The solubility of epothilones in water is one of its primary advantages over Taxol. Taxanes are weakly soluble, so usually given with solvents polysorbate 80 [32] and CremophorEL, that seriously lead to hypersensitivity and impair cardiac function [33]. Paclitaxel triggers macrophage-mediated activation, leads to creation of pro-inflammatory cytokines and nitric oxide, while epothilones do not have endotoxin action [34]. Epothilones have been approved to treat locally advanced, aggressively metastatic breast cancer [34, 35]. Although the superior pharmacokinetic properties of Epothilone in treatment of various drug-resistant tumors, however, the availability and cost remains the major challenges especially in the developing countries, that halts the application of this drug. To overcome the slower growth rate and physiological challenges by *S. cellulosum*, the epothilone biosynthetic potency by *Aspergillus fumigatus* raise the expectation for industrial production of Epothilone [9, 12].

The structural identity of the *A. fumigatus* Epothilone B was verified from FT-IR and LC–MS/MS analyses as consistent with our previously results [9, 12]. The cytotoxicity of the extracted Epothilone B from *A. fumigatus* was assessed for the different cell lines HCT-116, HepG-2, PC3, MCF-7, related to the control. From the IC_50_ values, Epothilone B of *A. fumigatus* had the highest activity against towards the HepG2 (6.32 μM), HCT 116 (7.34 μM), Pc3 cells (7.6 μM), compared to the Vero cells (18.7 μM) [2, 36]. Ixabepilone was recorded as a potent cytotoxic compound for the multidrug-resistant cells [37]. The anti-tubulin polymerizing potency of *A. fumigatus* epothilone was evaluated, with the IC_50_ value 0.45 μg/ml, related to the authentic antimitotic drug “Paclitaxel” [38]. The maximum arrest of the HepG-2 cells was informed at the G2/M, due to *A. fumigatus* epothilone*,* related to the normal cells. The percentage of cellular proliferation of the HepG-2 cells was reduced into 7.2% in response to addition of the IC25 of Epothilone compared to normal cells (14.9%), revealing the proficiency of the compound to combat the growth at the mitotic stage G2/M phase.

A significant induction to the cellular apoptosis was detected due to sample*,* related to the control*.* The total apoptosis percentage of the HepG2 cells was 26.5%, with the epothilone of *A. fumigatus*. Thus, upon addition of the target compound, the apoptosis was augmented by 12 folds, regularized to control, ensuring the biochemical significant effect of epothilone in inducing the cellular machinery of apoptosis [39, 40]. Epothilone B induces the apoptosis in a dose dependent-manner [41], by inducing chromatin condensation, and apoptotic bodies formation [37, 42]. The caspase-3 and caspase-9 activity in the HepG-2 cells cured with extracted Epothilone B was measured. The caspas-3 and caspase-9 activity was dramatically increased by 4 and 2.5 folds, respectively, with Epothilone B, related to the control cells. As well as, the molecular expressions of caspase-3 and 9 were increased by 1.3 and 1.5 folds, respectively, compared the control. An obvious increase on the generation of ROS level with Epothilone was observed. The ROS levels were increased by 40% in response to 0.4 μg/ml of Epothilone over the control. ROS generation has been used frequently as marker for numerous biological processes, DNA damage, and apoptosis. The LDH diffusion of tumor cells was increased exponentially with Epothilone in a concentration-dependent manner that might be ascribed to the partial rupture of plasma membrane, increasing their permeability.

Docking simulations were carried out to understand the interaction of epothilone with the target tubulin (5 W3 J) [26], compared to Taxol as reference drug. The docking result of the Epothilone B showed three hydrogen bonds with threonine (Thr 175), asparagine (Asn 204), and aspartic acid (Asp 177) of β-tubulin protein, with binding energy −9.96 kcal/mol, that was higher than Taxol (−7.87 kcal/mol), with the hydrogen bond as the most common interaction.

In conclusion, Epothilone B was extracted and chemically verified from *A. fumigatus.* The purified Epothilone B had a potent efficiency for the different cell lines, regarding to the normal cells. The purified compound has a strong anti-tubulin polymerizing activity by 2 folds, higher than Taxol, ensuring the efficiency to bind with the β-tubulin and stopping their polymerization. The purified Epothilone has a strong activity to induce the apoptosis and arresting cell cycle of HepG-2 at the G2-M phase. Additionally, the extracted Epothilone B induced the apoptotic response in HepG-2 cells by triggering the apoptotic genes of caspase-3 and 9, and formation of ROS and causing leakage of the LDH in the HepG2 cells.

## Data Availability

All datasets generated for this study are included in the article.
